# Association of Left Atrial Volume With Mortality Among ESRD Patients With Left Ventricular Hypertrophy Referred for Kidney Transplantation

**DOI:** 10.1053/j.ajkd.2009.12.033

**Published:** 2010-06

**Authors:** Rajan K. Patel, Alan G.M. Jardine, Patrick B. Mark, Anthony F. Cunningham, Tracey Steedman, Joanna R. Powell, Emily P. McQuarrie, Kathryn K. Stevens, Henry J. Dargie, Alan G. Jardine

**Affiliations:** 1 BHF Glasgow Cardiovascular Research Centre, University of Glasgow, UK; 2 Department of Renal Medicine, Western Infirmary, Glasgow, UK; 3 Department of Cardiology, Western Infirmary, Glasgow, UK

**Keywords:** End-stage renal disease, left ventricular hypertrophy, left atrial volume, cardiovascular magnetic resonance imaging (MRI)

## Abstract

**Background:**

Left ventricular hypertrophy (LVH) is common in patients with end-stage renal disease (ESRD) and an independent risk factor for premature cardiovascular death. Left atrial volume (LAV), measured using echocardiography, predicts death in patients with ESRD. Cardiovascular magnetic resonance (CMR) imaging is a volume-independent method of accurately assessing cardiac structure and function in patients with ESRD.

**Study Design:**

Single-center prospective observational study to assess the determinants of all-cause mortality, particularly LAV, in a cohort of ESRD patients with LVH, defined using CMR imaging.

**Setting & Participants:**

201 consecutive ESRD patients with LVH (72.1% men; mean age, 51.6 ± 11.7 years) who had undergone pretransplant cardiovascular assessment were identified using CMR imaging between 2002-2008. LVH was defined as left ventricular mass index >84.1 g/m^2^ (men) or >74.6 g/m^2^ (women) based on published normal left ventricle dimensions for CMR imaging. Maximal LAV was calculated using the biplane area-length method at the end of left ventricle systole and corrected for body surface area.

**Predictors:**

CMR abnormalities, including LAV.

**Outcome:**

All-cause mortality.

**Results:**

54 patients died (11 after transplant) during a median follow-up of 3.62 years. Median LAV was 30.4 mL/m^2^ (interquartile range, 26.2-58.1). Patients were grouped into high (median or higher) or low (less than median) LAV. There were no significant differences in heart rate and mitral valve Doppler early to late atrial peak velocity ratio. Increased LAV was associated with higher mortality. Kaplan-Meier survival analysis showed poorer survival in patients with higher LAV (log rank *P* = 0.01). High LAV and left ventricular systolic dysfunction conferred similar risk and were independent predictors of death using multivariate analysis.

**Limitations:**

Only patients undergoing pretransplant cardiac assessment are included. Limited assessment of left ventricular diastolic function.

**Conclusions:**

Higher LAV and left ventricular systolic dysfunction are independent predictors of death in ESRD patients with LVH.

Patients with end-stage renal disease (ESRD) have an increased risk of premature cardiovascular disease.^1^ Echocardiography has identified abnormalities in left ventricular structure and function—left ventricular hypertrophy (LVH), left ventricular systolic dysfunction (LVSD), and left ventricular dilation—that independently confer a poorer prognosis. These changes are common and have been termed “uremic cardiomyopathy.”^2,3^

LVH is present in approximately 67% of patients with ESRD^4^ and is the most common manifestation of uremic cardiomyopathy. Moreover, it is an independent risk factor for sudden cardiac death, heart failure, and cardiac arrhythmias in both the general population and patients receiving hemodialysis.^3,5^ Although common, the presence of LVH alone has a variable prognosis. Furthermore, reversal of LVH in patients with ESRD has proven difficult,^6,7^ and attempts have been made to identify additional abnormalities that predict death and are amenable to intervention.

Previous studies measuring left ventricular mass index (LVMi: defined as left ventricular mass corrected for body surface area [BSA]) in patients with ESRD have used echocardiography. However, estimation of LVMi is inaccurate because of changes in intravascular volumes during the inter- and intra-dialytic period and during dialysis and geometric assumptions that rely on intraventricular diameter to calculate LVMi using conventional echocardiography.^8^ Cardiovascular magnetic resonance (CMR) imaging provides detailed volume-independent measurement of cardiac structure and is considered the most accurate method for assessing ventricular dimensions in patients, including those with stage 5 chronic kidney disease.^9,10^

Left atrial dilation (corrected for BSA or height) measured using echocardiography is an independent predictor of mortality in the general population, and in patients with hypertension and ESRD.^11,12^ Causes of increased left atrial volume (LAV) in patients with ESRD include mitral valve disease, fluid overload, and impaired left ventricular diastolic relaxation and filling.^13^ LAV can be reliably and reproducibly measured using echocardiography and CMR imaging using the biplane area-length method.^14,15^ To this end, we postulated that increased LAV conferred poorer prognosis in ESRD patients with LVH. The aim of this study was to identify the prognostic effect of cardiac abnormalities, particularly increased LAV, in a cohort of ESRD patients with LVH identified using CMR imaging.

## Methods

### Study Design

This was a single-center prospective observational study to assess the determinants of all-cause mortality, particularly LAV, in a cohort of patients with ESRD with LVH defined using CMR.

### Setting

The Renal Transplant Unit at the Western Infirmary, Glasgow, provides transplant services to a population of 2.8 million people in the West of Scotland.^16^ The transplant waiting list has 300 patients at any time; approximately 120 new patients are waitlisted and approximately 80 adult transplants are performed annually.

### Participants

Since 2002,^17^ we have used CMR imaging as part of the standard assessment of patients who are referred for cardiovascular assessment before their inclusion on the kidney transplant waiting list. These patients were referred for pretransplant assessment because of advancing age or past/current history of cardiovascular disease.

All patients receiving maintenance hemodialysis therapy from our unit were studied on a nondialysis day with the aim to perform all investigations at the individuals' “dry weight.” Only patients with evidence of LVH on CMR imaging were entered into the study. To ensure that only nonvalvular causes of left atrial dilation were assessed, patients with mild to severe mitral valve disease on echocardiography, based on American Society of Echocardiography guidelines,^18^ were excluded from the study. In addition, all patients were in sinus cardiac rhythm at the time of scanning.

### Variables

All-cause mortality was the principle outcome in this study. To characterize factors associated with death, demographic information, past clinical history (at time of CMR scanning), and CMR measurements were recorded.

### CMR Technique and Analysis

Non–gadolinium-enhanced CMR imaging was performed using a 1.5-Tesla magnetic resonance imaging scanner (Sonata; Siemens Medical, www.medical.siemens.com) to assess LVM and function.^9,10^ Scans were performed on the day after their hemodialysis session. A fast imaging with steady-state precession (true FISP) sequence was used to acquire cine images in long-axis planes (vertical long axis, horizontal long axis, and left ventricular outflow tract) followed by sequential short-axis left ventricular cine loops (8-mm slice thickness, 2-mm gap between slices) from the atrioventricular ring to the apex. Imaging parameters, which were standardized for all participants, included the following values: repetition time, 3.14 ms; echo time, 1.6 ms; flip angle, 60°; voxel size, 2.2 × 1.3 × 8.0 mm; and field of view, 340 mm.

### Assessment of LVM and LAV Using CMR Imaging

LVM and LAV were analyzed by 2 observers (R.K.P. and A.G.M.J.) blinded to patient clinical characteristics. LVM was measured from short-axis cine loops using manual tracing of epicardial and endocardial end-systolic and end-diastolic contours, calculated using analysis software (Argus; Siemens Medical), and indexed for BSA (thus providing LVMi). According to established normal values,^19^ LVH was defined as LVMi >84.1 g/m^2^ (male) or >76.4 g/m^2^ (female). LVSD was defined as left ventricular ejection fraction <55%, and left ventricular dilation was defined as end-diastolic volume (EDV)/BSA >111.7 mL/m^2^ (male) or >99.3 mL/m^2^ (female) or end-systolic volume (ESV)/BSA >92.8 mL (male) or >70.3 mL (female).

The biplane area-length method for ellipsoid bodies was used to measure LAV.^14^ Horizontal and vertical long-axis cine images were used to obtain images of the left atrium at maximal filling. Atrial lengths and areas were measured from both views, and LAV was calculated. LAV was corrected for BSA (LAV/BSA). Left atrial appendages were included in these measurements.

### Mitral Valve Inflow Doppler Velocity Measurement

Echocardiography was performed by an experienced echocardiographer (A.F.C.) using an Acuson Sequoia C512 machine (Siemens Medical). Diastolic function was assessed using pulsed-wave Doppler^20,21^ from apical 4-chamber views to measure the ratio of early (E) to late (A) mitral inflow peak flow velocity (E:A ratio).

### Statistical Analysis

Data are described as mean ± standard deviation for normally distributed data) or median and interquartile range (IQR) for non-normal data. Survival data including survival time (mean ± SD) are shown as Kaplan-Meier graphs (with statistical comparison using log-rank test). These data were also analyzed using Cox multivariate survival analysis to assess the influence of multiple clinical and cardiac variables on outcome. Variables identified as significantly influential on outcome by univariate analysis were entered into a backward stepwise regression model. All analyses were performed using SPSS, version 15.0 (SPSS Inc, www.spss.com).

## Results

### Participants and Clinical Parameters

From 312 patients with ESRD assessed for kidney transplantation^17^ between 2002 and 2008, we identified 201 patients with LVH. Median follow-up was 3.62 years (IQR, 1.2-5.2) and transplant-censored follow-up was 1.69 years (IQR, 1.0-3.9). Mean age of patients was 51.6 ± 11.8 years and 72.1% were men. The first column in Table 1 lists renal replacement therapy mode, medical history, and cardiac drug history of the cohort.

**Figure 1:**
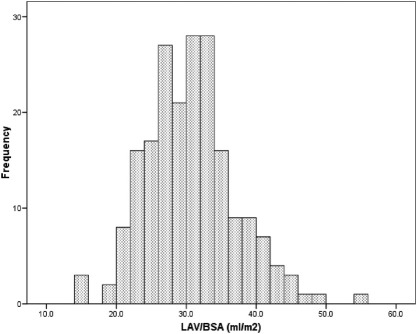
Frequency distribution of left atrial volume corrected for body surface area (LAV/BSA).

Seventy-one patients received a kidney transplant during the study period. There were 54 (26.9%) deaths during a 6.65-year follow-up period. Eleven deaths occurred after kidney transplantation. Overall survival at 12, 24, 36, 48, and 60 months was 92%, 87%, 79%, 77%, and 74%, respectively. Median LAV/BSA was 30.4 mL/m^2^. The distribution of LAV/BSA for this cohort is shown in Fig 1. There was no significant correlation between LVMi and LAV/BSA (*r* = 0.03; *P* = 0.7).

To identify determinants and consequences of increased LAV, we divided patients into high LAV (LAV/BSA equal to or higher than median; n = 100) or low LAV (LAV/BSA less than median value; n = 101; Table 1). High LAV was significantly associated with treatment with statins. Low LAV was significantly associated with male sex. There was no significant difference in age, number of patients who underwent kidney transplantation, BSA, and renal replacement type or duration between the high- and low-LAV groups. Furthermore, there was no significant difference in number of patients with diabetes mellitus, smoking history, and cardiovascular medical history (namely ischemic heart disease, chronic heart failure, cerebrovascular and peripheral vascular diseases). On comparison of cardiac medications, there were no other significant differences between the low- and high-LAV groups.

### Cardiac Parameters

Examining the entire cohort (Table 2), mean heart rate during CMR imaging was 77 ± 26 beats/min. Mean ejection fraction was 63.1% ± 14.4%, LVMi was 117.3 ± 31.1 g/m^2^, EDV/BSA was 86.3 ± 31.4 mL/m^2^, and ESV/BSA was 34.1 ± 25.3 mL/m^2^. Fifty (24.9%) patients had LVSD and 49 (24.4%) had left ventricular dilation. Doppler mitral valve inflow velocity measurement showed a mean peak E wave velocity of 0.74 ± 0.2 cm/s, mean peak A wave of 0.75 ± 0.2 cm/s, and E:A ratio of 1.04 ± 0.5.

We also compared structural CMR imaging and mitral valve echocardiography Doppler results between the low- and high-LAV groups. There were no significant differences between heart rate during CMR imaging, ejection fraction, left ventricular myocardial mass, EDV/BSA, ESV/BSA, and number of patients with LVSD or left ventricular dilation between groups. As a basic marker of diastolic function, Doppler mitral valve inflow velocity measurement showed no difference in E:A ratios between the low- and high-LAV groups.

### Cardiac Structure and Outcome

**Figure 2:**
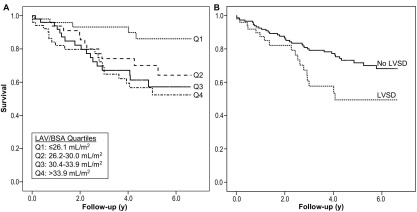
Kaplan-Meier survival curves according to (A) left atrial volume (LAV) corrected for body surface area (BSA) quartiles (Qs; median survival in years: Q1, 6.1 ± 1.7; Q2, 5.2 ± 2.5; Q3, 4.8 ± 2.6; Q4, 4.6 ± 2.6; log-rank *P* = 0.01) and (B) presence of left ventricular systolic dysfunction (LVSD; no LVSD group: n = 151, mean survival 5.4 ± 1.8 years; LVSD group: n = 50, 4.4 ± 3.9 years; log-rank *P* = 0.02).

We initially examined the effect of left atrial and ventricular abnormalities on patient survival. Divided into quartiles, increasing LAV was significantly associated with higher mortality: quartile (Q)1, 5 (10.0%) deaths; Q2, 13 (26.0%) deaths; Q3, 17 (34.0%) deaths; and Q4, 19 (37.3%) deaths; *P* = 0.01. Furthermore, increasing LAV was associated significantly with poorer prognosis (Fig 2A: *P* = 0.01).

Similarly, LVSD was associated with a significant reduction in mean survival time (Fig 2B: *P* = 0.02). Left ventricular dilation was associated with a non-significant decrease in patient survival (no left ventricular dilation, 5.2 ± 1.9 years vs left ventricular dilation, 4.7 ± 3.7 years; *P* = 0.2).

### Survival Analyses

Table 3 lists univariate and multivariate Cox survival analyses for patient clinical and cardiac characteristics. Univariate analyses showed that LVSD, LAV/BSA, and history of ischemic heart disease were significantly associated with death. Kidney transplantation was associated with a significant survival benefit. Advancing age increased the risk of death, but this did not reach statistical significance. Multivariate analysis (Table 3) was performed and showed LVSD, LAV/BSA, and history of ischemic heart disease as independent predictors of mortality. Kidney transplantation was independently associated with decreased mortality.

## Discussion

Patients with ESRD have an increased risk of premature cardiovascular mortality.^1^ In contrast to the general population, sudden, presumed arrhythmic, cardiac death, rather than myocardial infarction or heart failure, is the most common cause of death.^22,23^ Modification of traditional risk factors (eg, dyslipidemia) does not alter prognosis significantly,^24,25^ and reversal of LVH (the most common abnormality of uremic cardiomyopathy) is difficult.^6^ Alternative, potentially reversible, myocardial abnormalities have been sought to provide a target for intervention that may decrease cardiovascular death in this patient population.

Against this background, we prospectively assessed the effect of additional myocardial abnormalities and clinical history on survival in a cohort of ESRD patients with LVH. In particular, we investigated the prognostic effect of LAV, which has been shown previously using echocardiography as an independent predictor of death in dialysis patients.^26^ LAV can be calculated reliably from 2-dimensional echocardiography and CMR measurements.^14,15^ We restricted this study to patients with LVH to identify other potentially modifiable characteristics that predict death in patients with ESRD and established uremic cardiomyopathy.

Elevated LAV (higher than the median) was less common in male patients. This differs from previous studies that have shown removal of sex-related differences in LAV when corrected for body size. Neither sex nor BSA had an effect on survival in our analyses. There was no significant difference in cardiovascular disease history between groups (greater or less than median LAV). Furthermore, heart rate, LVEF, LVMi, and left ventricle chamber size (at end-diastole and end-systole) were similar in both groups. This suggests that left atrial size was not a marker of reduced diastolic filling time or impaired left ventricular systolic emptying.

In the survival analysis, higher LAV was significantly associated with poorer survival (Fig 2A). Multivariate analysis also showed that increased LAV/BSA and presence of LVSD independently predicted death in ESRD patients with LVH. As previously demonstrated,^17^ a clinical history of ischemic heart disease was a significant independent predictor of death, and kidney transplantation was independently associated with significantly improved survival.^27^

These data confirm previous studies investigating LAV and survival.^26^ However, in previous studies no analyses were performed in patients with pre-existing myocardial abnormalities. We believe that the strength of this study lies with accurate assessment of LVMi using CMR imaging, which provides an accurate, volume-independent, and reproducible method of measuring LVM.^9^

In the present study, LAV was not significantly correlated with LVM, suggesting that increased LAV is not caused solely by impaired atrial emptying into a large, poorly compliant, left ventricle. In other patient populations, increased LAVs are considered to reflect the long-term effects of increased ventricular filling pressures.^28^ Thus, when filling pressures are increased, the atria (like the ventricles) will enlarge in response to pressure and chronic volume overload.^29^ LAV was not associated with diastolic dysfunction in our population as measured using E:A ratio. We did not obtain tissue Doppler or pulmonary venous blood flow velocity data to fully evaluate diastolic function, and it is likely that diastolic dysfunction is common in ESRD patients with LVH. Thus, we postulate that in ESRD patients with LVH, left atrial enlargement is caused largely by diastolic dysfunction and also chronic fluid overload due to expansion in intravascular volume.

A decrease in LAV has been achieved in patients with mitral valve disease and atrial fibrillation^30,31^; however, its effect on overall prognosis is unknown. Whether tight control of fluid volume status in patients with ESRD similarly decreases LAV and mortality will require a controlled clinical trial.

We have previously shown that LVSD is associated with (often asymptomatic) ischemic heart disease and, in turn, poorer survival.^4^ This presumably is caused by occlusive large-vessel disease and inadequate growth of penetrating epicardial vessels in response to cardiac myocyte hypertrophy. Ventricular action potential propagation and recovery are impaired in the presence of LVH and LVSD, increasing the risk of ventricular re-entrant tachyarrhythmias.^32,33^ The prognostic benefits of improving systolic function of ESRD patients, using pharmacological approaches or modification of dialysis remain to be assessed.

In contrast to previous studies using echocardiography to assess left ventricular dimensions,^34^ higher LVMi was not a significant predictor of mortality in this cohort of patients. We have previously shown inaccuracies of echocardiography measurements in patients with ESRD,^35^ and it is likely that the use of CMR imaging to accurately assess myocardial mass will provide more reliable prognostic information in the future.

We accept that this study has some limitations. Patients recruited to this study were being assessed for kidney transplantation and may not be representative of all patients with ESRD. However, since these patients were considered healthy enough to be considered for a kidney transplant, we believe these results would be relevant to other patients with more significant comorbid conditions. In addition, we obtained limited information regarding ventricular diastolic function in our cohort and hopefully, as more detailed methods (eg, tissue Doppler) are utilized, these data will become available.

In conclusion, in ESRD patients with LVH, increased LAV and the presence of LVSD are independent predictors of death and may provide novel factors that may be amenable to modification to improve cardiovascular prognosis.

## Figures and Tables

**Table 1:** Clinical Information for the Entire Cohort and by Low or High LAV/BSA

Variable	Total (N = 201)	Low LAV/BSA (n = 101)	High LAV/BSA (n = 100)	*P*
Kidney transplants	71 (35.3)	40 (39.6)	31 (31)	0.2
Age (y)	51.6 ± 11.8	50.4 ± 12.6	52.8 ± 10.9	0.1
Men	145 (72.1)	82 (81.2)	63 (63)	0.004
BSA (m^2^)	1.76 ± 0.2	1.77 ± 0.2	1.75 ± 0.2	0.8
LAV (mL)	56.3 ± 9.7	50.4 ± 6.8	62.3 ± 8.4	0.001
SBP (mm Hg)	136.9 ± 24.6	135.6 ± 26.2	138.4 ± 22.8	0.5
DBP (mm Hg)	80.9 ± 12.9	82.2 ± 13.5	79.3 ± 12.1	0.2
RRT time (HD and PD only) (y)	1.7 ± 4.3	1.5 ± 4.3	1.9 ± 4.4	0.5
RRT type				
HD	108 (53.7)	56 (55.4)	52 (52)	
PD	52 (25.9)	23 (22.8)	29 (29)	0.6
Predialysis	41 (20.4)	22 (21.8)	19 (19)	
Diabetes mellitus	121 (60.2)	61 (60.4)	60 (60)	0.9
Ischemic heart disease	54 (26.9)	24 (23.8)	30 (30)	0.4
Heart failure	12 (6.0)	6 (5.9)	6 (6)	0.9
Cerebrovascular disease	12 (6.0)	6 (5.9)	6 (6)	0.9
Peripheral vascular disease	16 (8.0)	6 (5.9)	10 (10)	0.3
Smoking				
Never	107 (53.2)	56 (55.4)	51 (51)	0.5
Current/ex	94 (26.9)	45 (22.8)	49 (31)	
EPO receptor agonist	151 (75.1)	72 (71.2)	79 (79)	0.1
Hemoglobin (g/dL)	11.5 ± 1.7	11.5 ± 1.7	11.3 ± 1.5	0.2
β-Adrenoceptor blocker	87 (43.3)	45 (44.6)	42 (42)	0.8
Aspirin	83 (41.3)	38 (37.6)	45 (45)	0.2
Warfarin	7 (3.5)	4 (4.0)	3 (3)	0.7
ACEi/ARB	54 (26.9)	28 (27.7)	26 (26)	0.9
Diuretic	60 (29.6)	31 (30.7)	29 (29)	0.9
Calcium channel blocker	58 (28.9)	33 (32.7)	25 (25)	0.3
α-Adrenoceptor blocker	21 (10.4)	13 (12.9)	8 (8)	0.3
Statin	83 (41.3)	35 (34.7)	48 (48)	0.05

*Note:* Values expressed as mean ± standard deviation and number (percentage). The low-LAV/BSA group includes individuals with LAV/BSA less than the median value; the high-LAV/BSA group includes individuals with LAV/BSA equal to the median value or greater. Tests of significance are *t* test and χ^2^. Abbreviations and definitions: ACEi/ARB, angiotensin-converting enzyme inhibitor/angiotensin II receptor blocker; BSA, body surface area; DBP, diastolic blood pressure; EPO, erythropoietin; HD, hemodialysis; LAV, left atrial volume; LAV/BSA, left atrial volume corrected for body surface area; PD, peritoneal dialysis; RRT, renal replacement therapy; SBP, systolic blood pressure.

**Table 2:** Cardiac Information for Entire Cohort and by Low or High LAV/BSA

Variable	Total (N = 201)	Low LAV/BSA (n = 101)	High LAV (n = 100)	*P*
Heart rate (beats/min)	76.8 ± 26.1	76.3 ± 26.1	77.4 ± 26.1	0.3
Ejection fraction (%)	63.1 ± 14.4	63.4 ± 13.9	62.8 ± 15.0	0.8
LVMi (g/m^2^)	117.3 ± 31.1	115.7 ± 29.8	119.0 ± 32.5	0.4
EDV/BSA (mL/m^2^)	86.3 ± 31.4	86.9 ± 30.5	85.8 ± 32.4	0.8
ESV/BSA (mL/m^2^)	34.1 ± 25.3	33.7 ± 25.0	34.5 ± 25.7	0.8
LVSD^a^	50 (24.9)	23 (22.8)	27 (27)	0.5
LV dilation	49 (24.4)	23 (22.8)	26 (26)	0.6
Peak E wave (cm/s)	0.74 ± 0.2	0.77 ± 0.2	0.71 ± 0.2	0.3
Peak A wave (cm/s)	0.75 ± 0.2	0.76 ± 0.3	0.72 ± 0.2	0.3
E:A ratio	1.04 ± 0.4	1.06 ± 0.4	1.02 ± 0.4	0.7

*Note:* Values expressed as mean ± standard deviation and number (percentage). The low-LAV/BSA group includes individuals with LAV/BSA less than the median value; the high-LAV/BSA group includes individuals with LAV/BSA equal to the median value or greater. Tests of significance are *t* test and χ^2^. Abbreviations and definitions: A wave, late filling velocity; E wave, early filling velocity; EDV/BSA, end-diastolic volume corrected for body surface area; ESV/BSA, end-systolic volume corrected for body surface area; LVMi, left ventricular mass index; LVSD, left ventricular systolic dysfunction.

a Assessed using cardiovascular magnetic resonance imaging (ejection fraction <55%).

**Table 3:** Results of Univariate and Multivariate Cox Regression Survival Analyses of ESRD Patients With LVH

	Univariate Analyses			Multivariate Analyses		
Variable	HR	95% CI	*P*	HR	95% CI	*P*
Kidney transplantation	0.25	0.12-0.49	<0.001	0.22	0.11-0.43	<0.001
Ischemic heart disease	1.88	1.09-3.23	0.02	2.73	1.57-4.77	<0.001
LAV/BSA (per mL/m^2^)	1.07	1.03-1.12	0.001	1.06	1.02-1.11	0.009
LVSD	1.98	1.12-3.50	0.02	1.77	1.02-3.15	0.05
Ejection fraction (per %)	0.98	0.97-1.00	0.1			
LVMi (per g/m^2^)	1.00	0.99-1.01	0.6			
Left ventricular dilation	1.48	0.83-2.63	0.2			
SBP (per mm Hg)	1.00	0.98-1.02	0.9			
DBP (per mm Hg)	0.99	0.95-1.03	0.8			
Age (per y)	1.02	0.99-1.05	0.08			
BSA (m^2^)	0.28	0.08-1.10	0.1			
Male sex	0.64	0.37-1.10	0.1			
RRT time (per y)	1.03	0.91-1.18	0.6			
Diabetes mellitus	0.83	0.48-1.41	0.5			
Heart failure	1.08	0.39-2.98	0.9			
Cerebrovascular disease	1.97	0.84-4.62	0.1			
Peripheral vascular disease	1.22	0.52-2.84	0.7			
Current/ex smoking^a^	1.56	0.91-2.68	0.1			

*Note:* All-cause mortality (n = 54) is the dependable variable. Abbreviations and definitions: BSA, body surface area; HR, hazard ratio; CI, confidence interval; DBP, diastolic blood pressure; LAV/BSA, left atrial volume corrected for body surface area; LVMi, left ventricular mass index; SBP, systolic blood pressure; LVH, left ventricular hypertrophy; LVSD, left ventricular systolic dysfunction; RRT, renal replacement therapy.

a Never smoking is the reference category.
